# Functional MRI to Assess Decision-Making Capacity of Older Adults With Dementia: A Proof of Concept Study

**DOI:** 10.1093/geroni/igab046.1959

**Published:** 2021-12-17

**Authors:** Thomas TANNOU

**Affiliations:** Besançon University Hospital, Besancon, France

## Abstract

Assessment of decision-making capacity is essential to respect older adult dignity, particularly concerning major decision such as ageing in place. To date, it is the clinician's assessment, based on a global analysis of his clinical evaluation and neuropsychological tasks, which enables decision-making assessment. Given the difficulty it represents, and the ethical and societal issues raised, the research question concerns the contribution of neuro-imaging technologies as an aid to the evaluation of decision-making capacity. We included in our proof-of-concept study 4 healthy older patients and 2 older patients with dementia (mild stage) followed in a memory clinic. Each of the participants completed neuropsychological tests with a focus on executive functions, anosognosia and judgemental skills. Next, they performed a decision-making task, the Balloon Assessment Risk Task (BART) in functional MRI, and, finally, they participated in a semi-structured interview completed with interview of their caregiver. For both patients, their referring geriatrician was questioned a priori on his assessment of their decision-making capacity. The results showed a common activation pattern in functional MRI between the patient considered competent in decision-making and the healthy subjects, unlike the patient who was not clinically competent. The qualitative analysis highlighted major anosognosia in both pathological situations, but decision-making in everyday life situations differed between the 2 patients. This study shows the feasibility, on a sensitive topic, to explore the potential contribution of functional neuroimaging and semi-directed interviews as tools. It also demonstrates the value of conducting mixed research, combining neurosciences and social science to explore complex clinical issues.

